# Genetic Regulation of N6-Methyladenosine-RNA in Mammalian Gametogenesis and Embryonic Development

**DOI:** 10.3389/fcell.2022.819044

**Published:** 2022-03-14

**Authors:** Yuguang Chang, Mingliang Yi, Jing Wang, Zhikun Cao, Tingting Zhou, Wei Ge, Zafir Muhammad, Zijun Zhang, Yanqin Feng, Zihui Yan, Massimo De Felici, Wei Shen, Hongguo Cao

**Affiliations:** ^1^ Anhui Province Key Laboratory of Local Livestock and Poultry Genetic Resource Conservation and Bio-breeding, College of Animal Science and Technology, Anhui Agricultural University, Hefei, China; ^2^ Key Laboratory of Animal Reproduction and Biotechnology in Universities of Shandong, College of Life Sciences, Qingdao Agricultural University, Qingdao, China; ^3^ Department of Biomedicine and Prevention, University of Rome Tor Vergata, Rome, Italy

**Keywords:** m^6^A, spermatogenesis, oogenesis, embryo development, epigenetics

## Abstract

Emerging evidence shows that m^6^A is the most abundant modification in eukaryotic RNA molecules. It has only recently been found that this epigenetic modification plays an important role in many physiological and pathological processes, such as cell fate commitment, immune response, obesity, tumorigenesis, and relevant for the present review, gametogenesis. Notably the RNA metabolism process mediated by m^6^A is controlled and regulated by a series of proteins termed writers, readers and erasers that are highly expressed in germ cells and somatic cells of gonads. Here, we review and discuss the expression and the functional emerging roles of m^6^A in gametogenesis and early embryogenesis of mammals. Besides updated references about such new topics, readers might find in the present work inspiration and clues to elucidate epigenetic molecular mechanisms of reproductive dysfunction and perspectives for future research.

## Introduction

**FIGURE 1:**
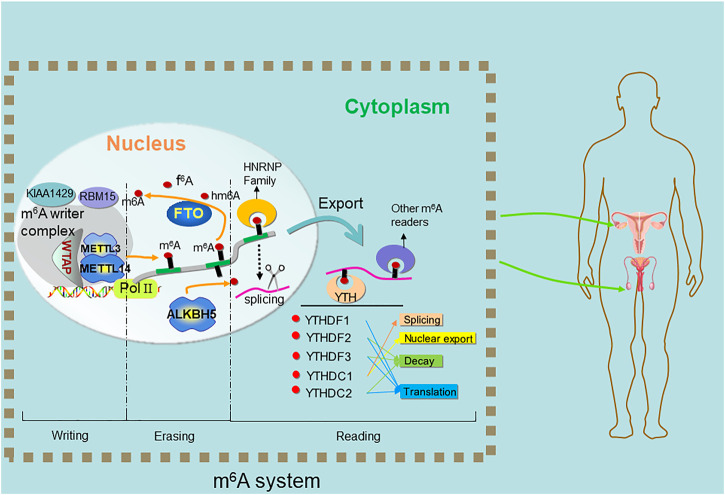
Methylation and demethylation of m^6^A on RNA. The *N* ^6^-methyladenosine (m^6^A) modification is imposed by a heterocomplex of two methyltransferases METTL3-METTL14 termed “writers”, assisted by WTAP. ALKBH5 and FTO, termed “erasers” catalyzes the direct removal of m^6^A. YTH family proteins termed “readers” recognize the m^6^A to carry out subsequent functions.

The emerging field of RNA modification is stimulating large-scale research. Compared with DNA methylation, RNA methylation is more diverse and widespread in various advanced organisms and widely affects various biological processes by regulating post-transcriptional gene expression. There are several key modifications in coding and non-coding RNA, including m^6^A (N^6^-methyladenosine), m^1^A (N^1^-methyladenosine) (Zhou et al., 2019), m^5^C (5- methylcytosine), m7G (N^7^-methylguanosine) (Malbec et al., 2019), 2′-O methylation, ac4C RNA acetylation, inosine and pseudouridine (Haruehanroengra et al., 2020). m^6^A is one of the most abundant chemical modifications in RNA discovered in 1975 (Schmidt et al., 1975) and the most abundant internal mRNA modification in eukaryotes (Roundtree et al., 2017a). In one third of the total mRNA of mammalian transcriptome, each mRNA has 3-5 m^6^A modifications (Dominissini et al., 2012). The m^6^A modification influences almost every step of RNA metabolism that comprises mRNA processing, mRNA export from nucleus to cytoplasm, mRNA translation, mRNA decay, and the biogenesis of long-non-coding RNA (lncRNA) (Pan, 2013) and microRNA (miRNA). The m^6^A occurs mostly in DRACH sequence (where D denotes A/G/U, R denotes A/G, and H denotes A/C/U) (Fu et al., 2014; Meyer et al., 2012), which is the m^6^A consensus motif. The m^6^A is enriched around stop codons, in 3ʹ untranslated regions (3ʹ UTRs) and within internal long exons and m^6^A occurs more in precursor mRNAs (pre-mRNAs). m^6^A is primarily regulated by three types of proteins, including methyltransferases (writers), demethylases (erasers) and methylated reading proteins (readers) (Table 1) (Figure 1).

**TABLE 1:** Functional roles of m^6^A regulators in RNA metabolism.

Type	m^6^A regulator	Function	References
m^6^A writer	METTL3	The catalytic subunit	Wang et al. (2016)
	METTL14	Activates METTL3 via allostery and recognition of RNA substrates	Wang et al. (2016)
	WTAP	Promotes METTL3-METTL14 heterodimer to the nuclear speckle	Ping et al. (2014)
	METTL16	Catalyzes m^6^A modification	Lan et al. (2019)
	KIAA1429	Regulates mRNA levels and alternative splicing of mRNA in oocytes	Hu et al. (2020)
	RBM15	Controls RNA splicing	Zhang et al. (2015)
m^6^A eraser	ALKBH5	Removes m^6^A methyl from RNA	Zheng et al. (2013)
	FTO	Regulates dynamic m^6^A modification and local translation of mRNA in axons	Yu J. et al. (2018), Mauer et al. (2017)
m^6^A reader	YTHDC1	Promotes RNA splicing and translocation	Kasowitz et al. (2018), Roundtree et al. (2017b)
	YTHDC2	Promotes translation and reduces mRNA abundance	Hsu et al. (2017), Wojtas et al. (2017)
	YTHDF2	Reduces mRNA stability	Huang et al. (2020)
	HNRNPA2B1	Promote pri-miRNA processing	Alarcón et al. (2015a)
	YTHDF1	Promotes protein synthesis	Wang et al. (2015)
	YTHDF3	Promotes mRNA translation	Li et al. (2017)
	IGF2BP1	Promotes the expression of SRF in a conserved and *N* ^6^-methyladenosine (m^6^A)-dependent manner	Müller et al. (2019)

Over the last decade, a variety of m^6^A detection methods have emerged. Among them, LC-MS (liquid chromatography-mass spectrometry) and ELISA (enzyme linked immunosorbent assay)/antibody-based colorimetry can detect the overall level of m^6^A on mRNA and conduct quantitative analysis. meRIP-seq (methylated RNA immunoprecipitation) (i.e., m^6^A-seq) and miCLIP-seq (m^6^A individual-nucleotide-resolution cross-linking and immunoprecipitation) are high-throughput sequencing methods, and m^6^A-seq can conduct qualitative analysis on hypermethylated mRNA regions, miCLIP-seq can accurately analyze the single base of m^6^A. In addition, as the third generation sequencing technology, nanopore-seq is mainly used for *de novo* genome assembly and can quantify differences in RNA modifications and detect different RNA modifications with position accuracy *in vitro* (Leger et al., 2021; Pratanwanich et al., 2021). ELISA-colorimetry, meRIP-seq and miCLIP-seq can only detect m^6^A indirectly via antibody binding, whereas mass-spectrometry and now RNA modification detection by Nanopore direct RNA-seq can detect m^6^A directly.

**FIGURE 2:**
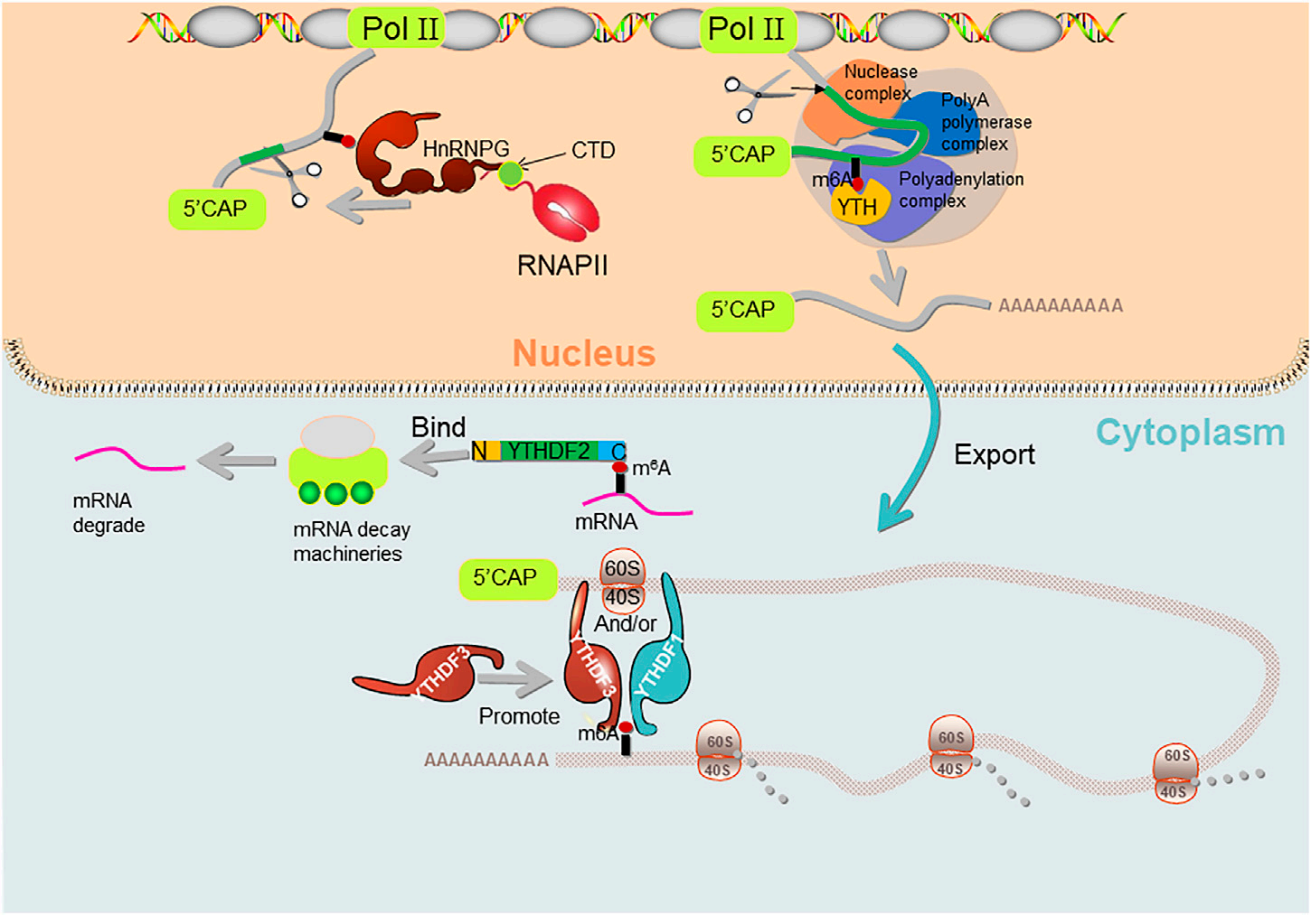
The molecular mechanisms of how m^6^A affects splicing/polyadenylation/translation. YTHDF1 and YTHDF3 regulate m^6^A both individually and together. hnRNPG binds to m^6^A-containing 3′-terminal mRNA and interacts with 40S and 60S ribosomal subunits to regulate mRNA translation, and YTHDF3 also facilitates this process. hnRNPG binds to m6a-containing pre-mRNA and interacts with RNA polymerase II (RNAP II) phosphorylated carboxy-terminal domain (CTD) of RNA polymerase II, which in turn regulates alternative splicing of mRNAs. C-YTHDF2 selectively recognizes m^6^A-containing mRNAs, while N-YTHDF2 on the other side binds to mRNA decay machineries and regulates mRNA degradation. YTH domain binds to the m^6^A-containing pre-mRNA and regulates the length of the 3′ UTR through polyadenylation complexes.

It has been confirmed that for most cells in reproductive and other systems, the precise regulation of gene expression occurs at the transcriptional, post-transcriptional and translation levels (Figure 2). Germ cells are a special type of cells in the body, which can carry out meiosis. The combination of haploid sperm and egg can give birth to new living individuals, so as to realize the transmission of genetic information. However, the mechanisms regulating the generation and development of these gametes in the reproductive system are not fully understood. Recent studies have shown that the epigenetic regulation of mRNA m^6^A modification plays an important role in gametogenesis of many species having crucial roles in meiotic initiation and progression as well as in many other processes unique to spermatogenesis and oogenesis.

In the present work, we review and discuss recent evidence indicating that m^6^A modification can regulate many processes of reproduction both in males and in females, and provide clues about epigenetic molecular mechanisms of reproductive dysfunction and perspectives for future research.

## The Role of m^6^A in Spermatogenesis

Spermatogenesis is an orderly, accurate and complex process, which mainly includes biological phenomena such as mitosis, meiosis, spermiogenesis, and spermiation. In mammals, spermatogonial stem cells (SSCs) undergo self-renewal mitotic divisions and at the same time give rise to A-paired (Apr) and A-aligned (Aal) spermatogonia. Aal spermatogonia can divide and proliferate to form A1 spermatogonia which generate type B spermatogonia. These enter into meiosis as primary spermatocytes after some mitotic divisions; at the end of the first meiotic division, each primary spermatocyte forms two secondary spermatocytes which in turn at the end of the second meiotic division, generate two haploid spermatids. Finally, these, through a complex process termed spermiogenesis, develop into spermatozoa which are released inside the lumen of seminiferous tubules (spermiation) (Griswold, 2016; O'Donnell et al., 2011). m^6^A modification of mRNA plays a key role in spermatogenesis and can ensure coordinated translation at different stages of spermatogenesis (Lin et al., 2017).

### Writers, Erasers and Readers of m^6^A as Regulators of mRNAs

The essential role that m^6^A plays in the regulation of spermatogonia mitosis, meiosis and spermiogenesis in mammals has been recently reviewed (Gui and Yuan, 2021) and is schematically summarized.

METTL3 (methyltransferase like 3) and METTL14, two of the methyltransferase “writers” of m^6^A, able to form stable heterodimers, are essential for spermatogenesis and fertility in male mice. In fact, for early male germ cells, knockout of METTL3 or METTL14 will destroy the quiescent state and lead to rapid depletion and complete loss of SSCs in testicular tissue (Lin et al., 2017; Xu et al., 2017). Transcriptome and m^6^A modification analysis showed that the expression and alternative splicing of many key genes in SSCs had changed. These results showed that METTL3 and METTL14 were necessary to control the proliferation and differentiation of SSCs by methylation of transcripts of key regulatory factors (Xu et al., 2017). Combined deletion of METTL3 and METTL14 in late germ cells of male mice, rather than removing a single gene, leads to spermatogenesis disorder in testis, but will not affect sperm meiosis. In particular, double gene knockout mice showed sperm flagellum defect, severe reduction of motility and abnormal sperm head, which was similar to human OAT (oligo-astheno-teratozoospermia) syndrome. At molecular level, haploid specific genes that are particularly important for spermatogenesis cannot be translated in the spermatids of these mutants (Lin et al., 2017). METTL3 is highly conserved across species, in fact, it has been shown that its inactivation prevented the embryo from completing the globular stage in *Arabidopsis* (Zhong et al., 2008), while in yeast, ablation of IME4 (a homolog of METTL3) led to the loss of the function of initializing meiosis and sporulation (Schwartz et al., 2013). In humans, it has been found that METTL3 plays key roles in increasing m^6^A content in sperm RNA and increased m^6^A content is a risk factor for asthenozoospermia and affects sperm motility (Yang et al., 2016).

In addition to the role in spermatogenic cells, m^6^A is involved in spermatogenesis by affecting the function of testicular somatic cells. The loss in Sertoli cells of WTAP, the regulatory subunit of the METTL3/METTL4 complex, impairs their ability to sustain the SSC niche (Jia et al., 2020).

The “erasers” of m^6^A FTO (fat mass and obesity-associated protein) and ALKBH5 are highly expressed in testis. Ablation of ALKBH5 in mice resulted in spermatogenesis defects, but the mice showed no other significant phenotypic changes (Zheng et al., 2013). On the other hand, the ablation of FTO in mice resulted in significant reduction of adipose tissue and weight loss, but there was no obvious symptom of spermatogenesis defect (Fischer et al., 2009). Nevertheless, Huang et al. (2018) reported that the knockout of FTO by CRISPR/cas9 technology resulted in chromosome instability and G2/M phase arrest in mouse GC-1 spermatogonial cell line. In wild-type cell lines, the expression of FTO partially saved the occurrence of this phenomenon, rather than demethylase inactivated FTO. In addition, the deletion of erasers significantly reduced the expression of mitotic checkpoint complexes and G2/M-related regulatory factors.

ALKBH5 knockout mice showed a remarkably lower breeding success rate and smaller testes than wild-type. The occurrence of these defects was closely related to the extensive apoptosis of germ cells and the abnormal morphology of seminiferous tubules, and spermatozoa were greatly reduced in number and displayed aberrant morphologies and reduced motility. At the molecular level, RNA-Seq analyses indicated aberrant expression of several genes in spermatogenic cells from ALKBH5-deficient mice (Zheng et al., 2013). In this regard, Tang et al. (2018) recently found that in the nuclei of mouse spermatocytes and round spermatids, ALKBH5-mediated m^6^A erasure is involved in the correct splicing of mRNA. At the same time, it also plays an important role in the production of longer 3′-UTR mRNA. Interestingly, ALKBH5 and FTO have been found in human testis, and there are two missense mutations, which may have potentially harmful effects on the function of FTO, as well as genetic variants of the proteins associated with altered semen quality, were reported (Landfors et al., 2016).

**FIGURE 3:**
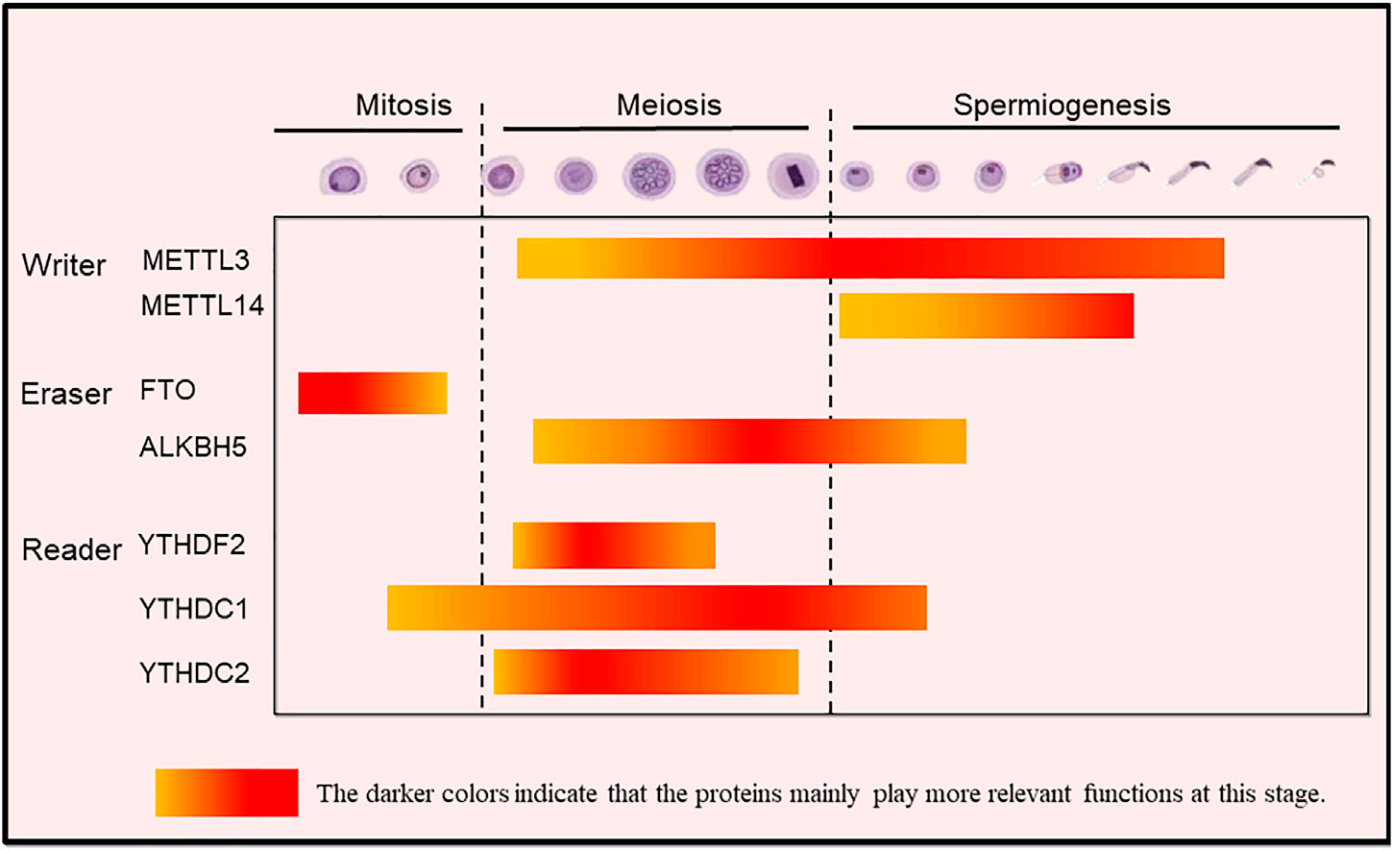
The m^6^A modification-related proteins exerting stage-dependent functions during spermatogenesis in mammals. Writers: METTL3 (and at lesser extent METTL14) regulates mainly spermatogonial homeostasis; METTL3/METTL14 complexes regulate spermiogenesis, namely the formation of flagellum; METTL16 inhibits spermatogonia proliferation and ensures normal germ cell differentiation; Erasers: FTO regulates cell-cycle in spermatogonia; ALKBH5 improves translation efficiency and rapid protein turnover mainly during spermiogenesis; Readers: YTHDF2 regulates meiotic progression; YTHDC1 is involved in co-transcriptional and/or post-transcriptional regulations in spermatogonia, spermatocytes, and round spermatids; YTHDC2 promotes translation and plays an essential role in gene expression and promotes translation during meiosis. The highlighted color indicates that the proteins mainly play functions at this stage.

Proteins termed “readers” recognize and bind m^6^A-RNA. Through binding single-stranded RBMs (RNA binding motif), YTHDF1, YTHDF2, YTHDF3, and YTHDC1 work in concert to quickly process mRNAs in the nucleus, control the translation of mRNAs, and then participate in the rapid degradation of translated mRNAs (Hsu et al., 2017). Among these, the synergistic interaction of YTHDF1 and YTHDF3 can regulate the translation of these mRNAs, YTHDF2 can accelerate the decay of mRNA, and YTHDC1 can participate in the nuclear processing of target mRNAs (Xiao et al., 2016; Shi et al., 2017). In addition, YTHDC2 recognizes and exerts post transcriptional control of different RNA targets through multiple RNA binding domains in YTHDC2 that function to improve translation efficiency and decrease their mRNA abundance. Like for the writers and erasers, studies focused on YTHDC2, YTHDC1 and YTHDF2 showed that they play crucial roles in spermatogenesis (Figure 3). YTHDC2 can bind to multiple transcripts, including cyclin A2 (Ccna2) and other mitotic transcripts, and can interact with RNA granule components. These phenomena indicate that YTHDC2 participates in the meiotic process of germ cells through post transcriptional regulation (Bailey et al., 2017). Knockout of YTHDC2 in mice leads to infertility and smaller testes and ovaries. In addition, germ cells cannot progress through the zygotic stage of prophase I of meiosis (Hsu et al., 2017; Wojtas et al., 2017). Similarly, in the testis of infertile “Ketu” mutant mice with missense YTHDC2 mutation, germ cells experienced a failed meiosis attempt and tried to start the expression of landmark meiotic proteins and DNA recombination, but still could not completely shut down the mitotic process of spermatogonia, resulting in premature entry into an abnormal metaphase like state, and enter the outcome of apoptosis (Jain et al., 2018). Abby et al. (2016) reported that during prophase I of meiosis, MEIOC, a meiosis specific protein, interacts with YTHDC2 in an RNA independent manner to stabilize transcripts. Soh et al. (2017) also found that MEIOC interacts with YTHDC2, but MEIOC may destroy its target transcripts to maintain the extension of meiotic prophase. Alternatively, Jain et al. (2018) suggested that YTHDC2 interacts with proteins that have known roles in nonsense-mediated mRNA decay such as XRN1, UPF1 and MOV10 rather than with MEIOC to destabilize its target transcripts. In addition, YTHDC2 regulates pachytene by maintaining meiotic transcriptome and preventing microtubule network changes that may lead to telomere aggregation in male meiosis (Liu et al., 2021). Therefore, YTHDC2 as a protein with multiple domains, has complex functions related to the regulation of translation efficiency and transcriptional stability.

**FIGURE 4:**
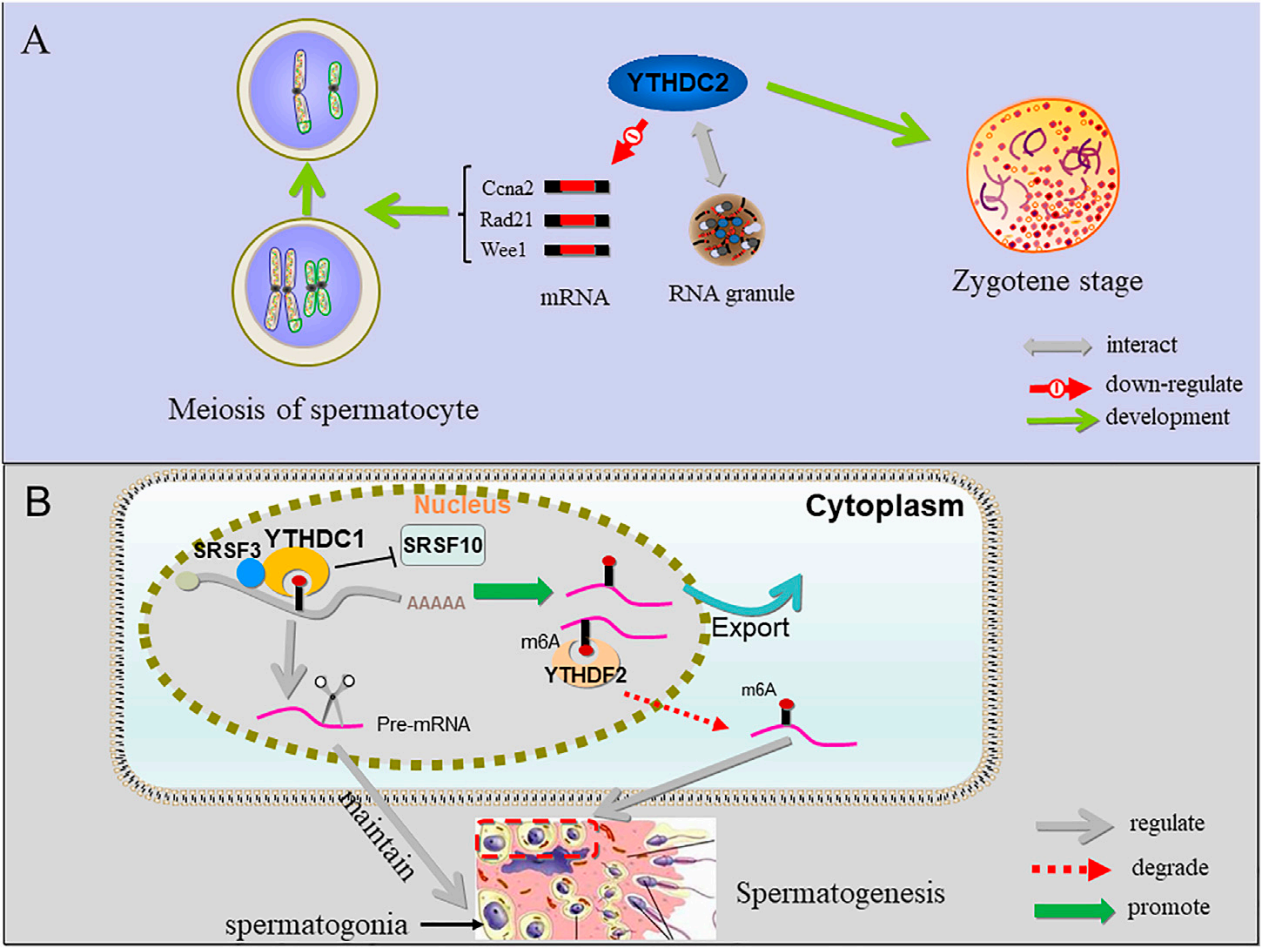
The function of YTHDC1, YTHDC2 and YTHDF2 in sperm cells. **(A)** YTHDC2 binds multiple transcripts including Ccna2 and other mitotic transcripts and interacts with RNA granule components, suggesting that proper progression of germ cells through meiosis is licensed by YTHDC2 through post-transcriptional regulation; when YTHDC2 is inactivated, spermatocytes do not pass the zygotene stage. **(B)** YTHDC1 can recruit SRSF3 and block SRSF10 to regulate alternative splicing of pre-mRNA crucial for maintaining spermatogonia. In addition, YTHDC1 can also promote the output of m^6^A-containing mRNA. YTHDF2 binds to the m^6^A site and degrade RNAs containing m^6^A involved in controlling migration and proliferation of spermatogonia.

YTHDC1 exists in the nuclei of spermatogonia, spermatocytes and round spermatids in the state of transcriptional activation, but not in elongating and elongated spermatids in which transcription is not activated. These results indicated that YTHDC1 participates in co-transcriptional and/or post transcriptional regulation during mitosis and meiosis (Kasowitz et al., 2018). YTHDC1, as a nuclear binding protein of m^6^A, by recruiting the pre-mRNA splicing factor srsf3 (SRp20), promote the targeting of the exon of the transcriptional product mRNA and block the binding activity of srsf10 (SRp38) mRNA, which plays an important role in maintaining the development of mouse spermatogonia (Xiao et al., 2016; Kasowitz et al., 2018). In fact, in YTHDC1 knockout mice, only sertoli cells remain in the seminiferous tubules. In addition, YTHDC1 can also promote the output of m^6^A-containing mRNA (Roundtree et al., 2017b) (Figure 4).

YTHDF2 is expressed in all stages of spermatogenesis, and it is highly expressed in pachytene spermatocytes. During spermatogenesis, YTHDF2 is cytoplasmic in both the germ and the somatic cells. male YTHDF2 knockout mice are fertile with normal seminiferous tubule histology and YTHDF2 knockout did not affect spermatogenesis *in vivo* (Ivanova et al., 2017), *In vitro*, mouse spermatogonia (GC-1 cells) after knockout of YTHDF2 showed down-regulation of the expression of MMPs (matrix metallopeptidases), affecting cell adhesion and proliferation (Huang et al., 2020). YTHDF2 binding leads to changes in the translation efficiency and stability of m^6^A-containing RNAs, causes degradation of m^6^A-containing RNAs and may affect the stability of m^6^A containing transcripts involved in controlling the migration and proliferation of spermatogonia (Du et al., 2016; Huang et al., 2020).

### Writers, Erasers and Readers of m^6^A as Regulators of Non-coding RNAs

**FIGURE 5:**
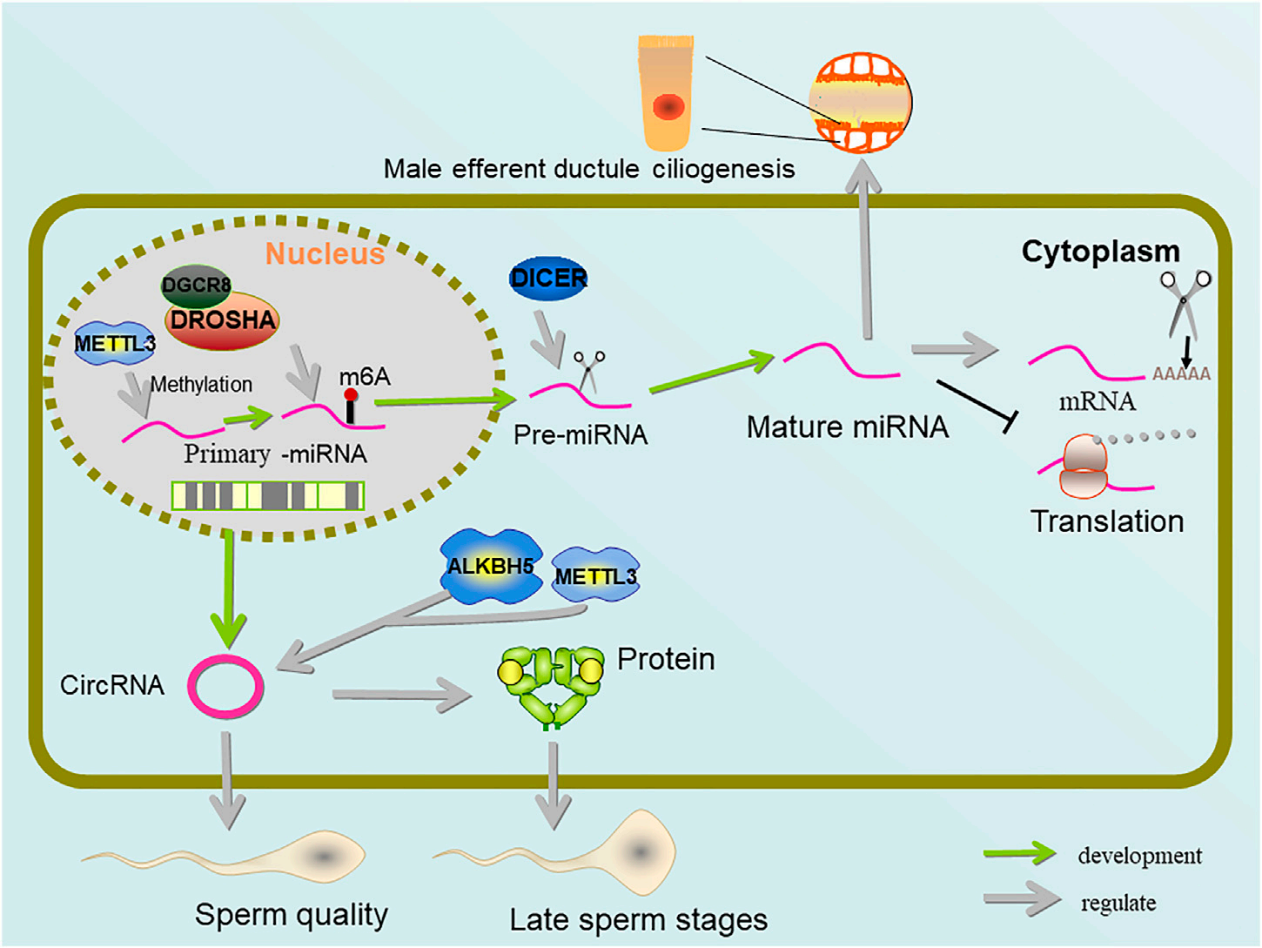
Regulation of miRNA and circRNA by METTL3 and ALKBH5. Pre-miRNA methylated by METTL3 shortens the mRNA poly(A) tail, inhibits mRNA translation, and forms output tubule cilia. METTL3 and ALKBH5 jointly control the amount of m^6^A on circRNA, which is important for sperm quality and protein supplementation in late sperm stages.

m^6^A modification is widely involved in the expression and regulation of transcripts. It is used not only to modify mRNA, but also to modify non-coding RNA, such as microRNAs (miRNAs). miRNA is a non-coding single stranded small RNA molecule with a length of about 21–23 nucleotides (Bartel, 2018). miRNA widely exists in eukaryotic cells. After the transcription of gene expression, miRNA accurately regulates the development of eukaryotic cells by means of translation inhibition and mRNA cleavage (Ambros et al., 2003). The processing of mature miRNAs involves two classical steps: In the nucleus, the primary miRNA (pri-miRNA) will be processed through the microprocessor complex composed of RNA binding protein DGCR8 and ribonuclease type III Drosha to form pre-miRNA, then transported to the cytoplasm through the nuclear pore, and further formed into mature miRNA by Dicer cutting (Bartel, 2009). miRNAs and siRNAs (small interfering RNAs), both of which are Argonaute-bound small RNAs, are essential for mammalian spermatogenesis (Hayashi et al., 2008). During the active transcription of meiotic genes in pachytene spermatocytes and round spermatocytes, miRNAs were significantly enriched (Ro et al., 2007; Kotaja, 2014). In addition, knocking out two miRNA clusters (mir-34B/C and-449A/B/C) will lead to the imbalance of expression of many key genes and the failure of multiciliogenesis in spermatogenic output tubules of mouse testis (Yuan et al., 2015; Yuan et al., 2019) (Figure 5). Moreover, piRNAs are a single stranded, 23–36 nucleotide (NT) RNA that serves as a guide for animal specific argonaute protein (piwi proteins), and a class of small RNA molecules necessary to maintain the integrity and fertility of germ cell lines (Bagijn et al., 2012). piRNAs are also involved in m^6^A regulation, YTHDC2 binds to multiple transcripts, including specific piRNA precursors, and regulates the meiotic process of germ cells (Bailey et al., 2017) (Figure 5).

During miRNA maturation, pri-miRNAs are methylated by METTL3, which enables it to be recognized by hnRNP A2/B1 (heterogeneous nuclear ribonucleoprotein A2/B1) and processed by DGCR8 (Alarcón et al., 2015a; Alarcón et al., 2015b). As an RNA binding protein and a potential m^6^A reader, sumoylated hnRNP A2/B1 has been shown to regulate the loading and sorting of m^6^A-modified miRNAs into exosomes (Villarroya-Beltri et al., 2013). In addition, hnRNP A2/B1 binding to pri-miRNAs has recently been proposed to occur via an “m6A switch” mechanism where m^6^A-induced RNA unfolding enhances adjacent hnRNP A2/B1 binding, thus promoting pr-miRNA processing (Wu et al., 2018). Thereby hnRNP A2/B1 binds to DGCR8 to promote pri-miRNA processing and affect miRNAs maturation (Alarcón et al., 2015a).

As another targets of METTL3, circRNAs, which are derived from the splicing of coding genes and contain an ORFs (open reading frames), are rich in testis and seminal plasma. During the process of spermatogenesis, circRNAs gradually become a regulator of sperm quality, particularly when late pachytene spermatocytes evolve into round and elongated sperm cells. It compensates for linear RNA degradation in late spermatogenesis and maintains protein levels during chromatin condensation (Chioccarelli et al., 2019; Tang et al., 2020). In addition, a large number of abnormally expressed circRNAs can be detected in patients with NOA (non-obstructive azoospermia), and the level of circRNAs is lower than normal (Ge et al., 2019; Gao et al., 2020). circRNAs belong to a class of covalently closed transcripts produced by back-splicing reaction. There is a significant direct correlation between METTL3 demand, YTHDC1 binding and the ability of m^6^A exon to back-splicing. m^6^A modification regulates its translation through the identification of YTHDF3 and eIF4G2 (Di Timoteo et al., 2020). At the same time, it was discovered that deleting METTL3 resulted in a large drop in circRNAs in the testis, which was compatible with METTL3’s identification as m^6^A methyltransferase, whereas deleting ALKBH5 also resulted in a significant decrease in spermatogenic cells (Tang et al., 2020).

## m^6^A RNAs in Oogenesis and Folliculogenesis

Oocyte development includes oogenesis, folliculogenesis and ovulation. Briefly, oogenesis mainly refers to differentiation of oogonia from primordial germ cells (PGCs), oogonia proliferation and entering into meiosis as primary oocytes, growth and cytoplasmic/nuclear maturation of oocytes. As primary oocytes reach the diplotene stage of meiotic prophase I, their inclusion within a primordial follicle imposes meiotic arrest and marks the beginning of folliculogenesis culminating in ovulation.

**FIGURE 6:**
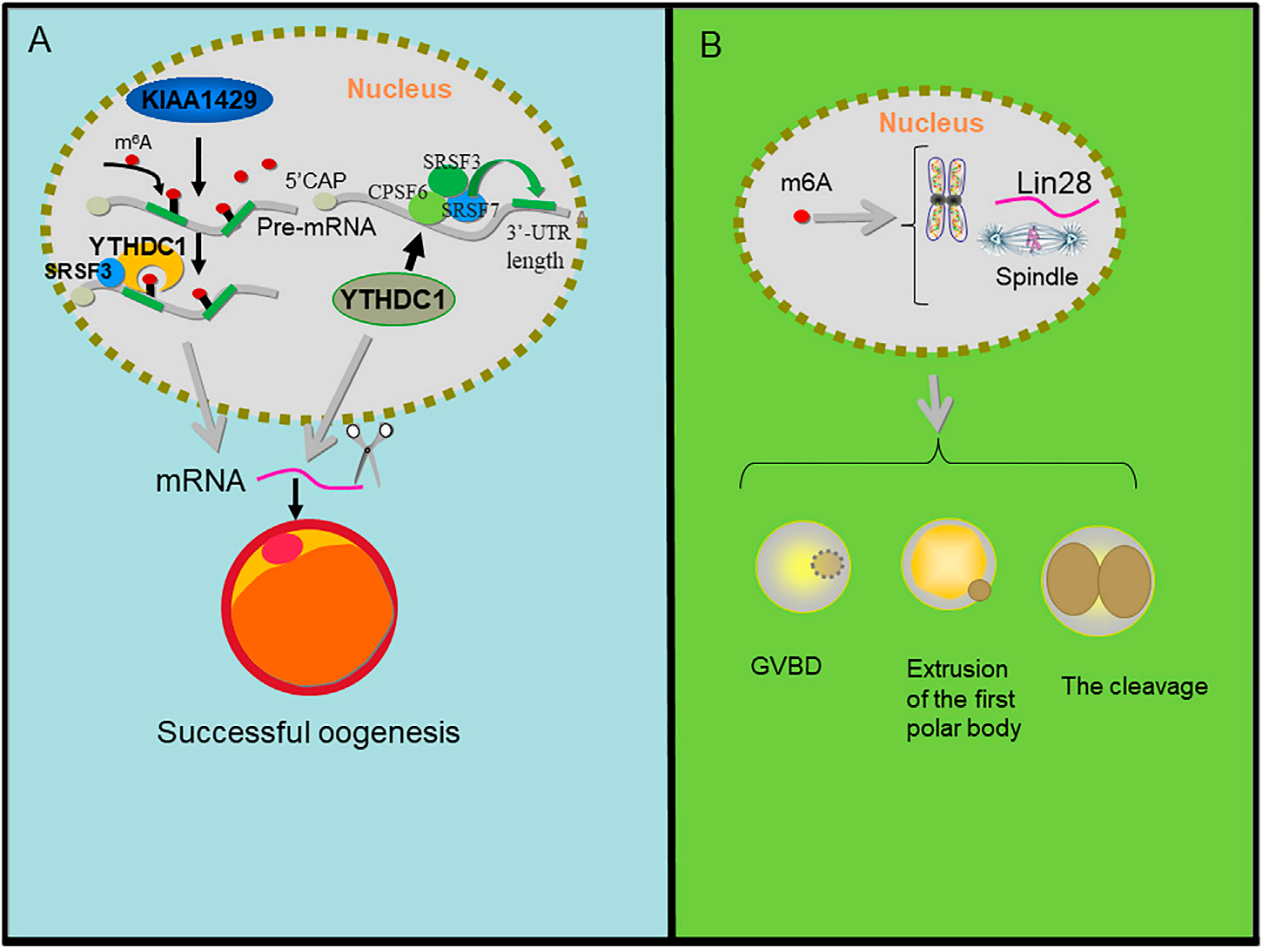
m6A regulation in oogenesis. **(A)** KIAA1429 mediates the m6A modification on pre-mRNA, then YTHDC1 recognizes the m^6^A signal and recruits SRSF3 to the binding regions on the KIAA1429-mediated pre-mRNA and YTHDC1 alters 3′ UTR length through associating with pre-mRNA 3′ end processing factors CPSF6, SRSF3, and SRSF7 which results in splicing of mRNA and then promotes oogenesis. **(B)** m^6^A affected gene abundance of pluripotent marker Lin28. It interferes with pluripotent regulation of chromosome/spindle tissue and regulates GVBD, PB1 extrusion, cleavage, and blastocyst development of parthenogenesis.

For mammalian oocytes, the germinal vesicles (GV) in the nucleus of oocytes gradually become inactive after a period of active transcription in the rapid growth stage (Bachvarova et al., 1985; De La Fuente et al., 2004). Under the condition of inactive transcription, the completion of meiosis and the development of early embryos largely depend on oocyte derived maternal RNA. Therefore, the regulation of oocyte gene expression is mainly based on mRNA stability and translation level. Increasing evidence indicates that also m^6^A-RNA is a crucial part of these regulatory processes.

m^6^A modification plays an important role in the development of oocytes and follicles (Figure 6). There are dynamic changes in m^6^A methylomes during the developmental transition from small to large sized follicles (Cao et al., 2020). The loss of m^6^A compromises gamete maturation and gametogenesis processes (Lasman et al., 2020; Fang et al., 2021). Among the key genes involved in m^6^A modification, METTL3, METTL14 and ALKBH5 were highly expressed in mouse oocytes and played important biological functions (Sui et al., 2020). The specific inactivation of the key m^6^A methyltransferase METTL3 in oocytes leads to the accumulation of DNA damage, follicular development defects and abnormal ovulation, and m^6^A modification through METTL3 affects oocyte meiosis (Mu et al., 2021). In fully grown mouse GV oocytes, METTL3 knockout seriously inhibits maturation and leads to abnormal MZT (maternal-to-zygotic transition) in the embryo, which may be caused by lowering mRNA translation efficiency of genes such as Cltc, Pcnt, Spdl-1 and Msy2 and/or interfering with their transcript degradation (Sui et al., 2020). In pig oocytes, METTL3, FTO, and WTAP elevated the transcription level and increased global m^6^A amount throughout meiotic maturation occurred. Reduced nucleic acid methylation impairs meiotic maturation and developmental potency of pig oocytes presumably due to reduced abundance of the pluripotency marker Lin28 and chromosome/spindle abnormalities (Wang et al., 2018). In addition, ascorbic acid was reported to reprogram the methylation status of not only DNA and histone, but also m^6^A in RNA, to improve pig oocyte maturation and developmental competence (Yu X.-X. et al., 2018).

KIAA1429 (VIRMA, vir-Like m^6^A methyltransferase associated), a component of the RNA m^6^A methyltransferase complex, plays critical roles in folliculogenesis and the maintenance of oocyte competence. The specific deletion of KIAA1429 in oocytes leads to female infertility, and fully grown GV (germinal vesicle) oocytes fail to undergo GVBD (germinal vesicle breakdown), so they lose the ability to resume meiosis. In addition, the loss of KIAA1429 could also lead to abnormal RNA metabolism in GV oocytes and the conditional depletion of KIAA1429 decreased the m^6^A levels in oocytes and mainly affected the alternative splicing of genes associated with oogenesis (Hu et al., 2020).

During oocyte maturation, the deletion of YTHDF2 leads to the inability to regulate the transcriptional amount of key oocyte development genes (Ivanova et al., 2017). YTHDC1 knockout oocytes are blocked at the primary follicular phase. In the oocyte nucleus, YTHDC1 plays a key role in the processing of pre-mRNA transcripts, which may be closely related to the 3′terminal processing modifiers CPSF6, SRSF3, and SRSF7 of pre-mRNA. In addition, the deletion of YTHDC1 leads to extensive selective polyadenylation in growing oocytes, which changes the length of 3′UTR in oocytes and leads to a large number of selective splicing defects (Kasowitz et al., 2018). Using conditional mutagenesis, conditional deletion of YTHDF2 leads to low oocyte development quality or low zygote development ability in mice, but leads to female specific infertility. YTHDF2 is autonomously required within the germline to produce MII oocytes that are competent to sustain early zygotic development. Oocyte maturation is associated with a wave of maternal RNA degradation, and the resulting relative changes in the MII transcriptome are integral to oocyte quality. The loss of YTHDF2 results in the failure to regulate transcript dosage of a cohort of genes during oocyte maturation, with enrichment observed for the YTHDF2-binding consensus and evidence of m^6^A in these upregulated genes. YTHDF2 is an intrinsic determinant of mammalian oocyte competence and early zygotic development (Ivanova et al., 2017). YTHDF2 exerts essential functions in the regulation of mammalian development during oocyte maturation and early zygotic development in female fertility (Zhao and He, 2017).

Finally, a large number of m^6^A modifications have been found in porcine granulosa cells, which may be related to steroids synthesis and follicle development (Cao et al., 2020). In the process of follicle selection that precedes ovulation, it was found that m^6^A is widely distributed in the follicular transcriptome, and methylation enrichment is negatively correlated with gene expression (Fan et al., 2019). In particular, it has been suggested that decrease of FGF transcripts mediated by m^6^A modification is required for correct granulosa cell differentiation and follicular selection (Yu et al., 2011; Lotz et al., 2013).

## m^6^A Regulation of Embryonic Development

**FIGURE 7:**
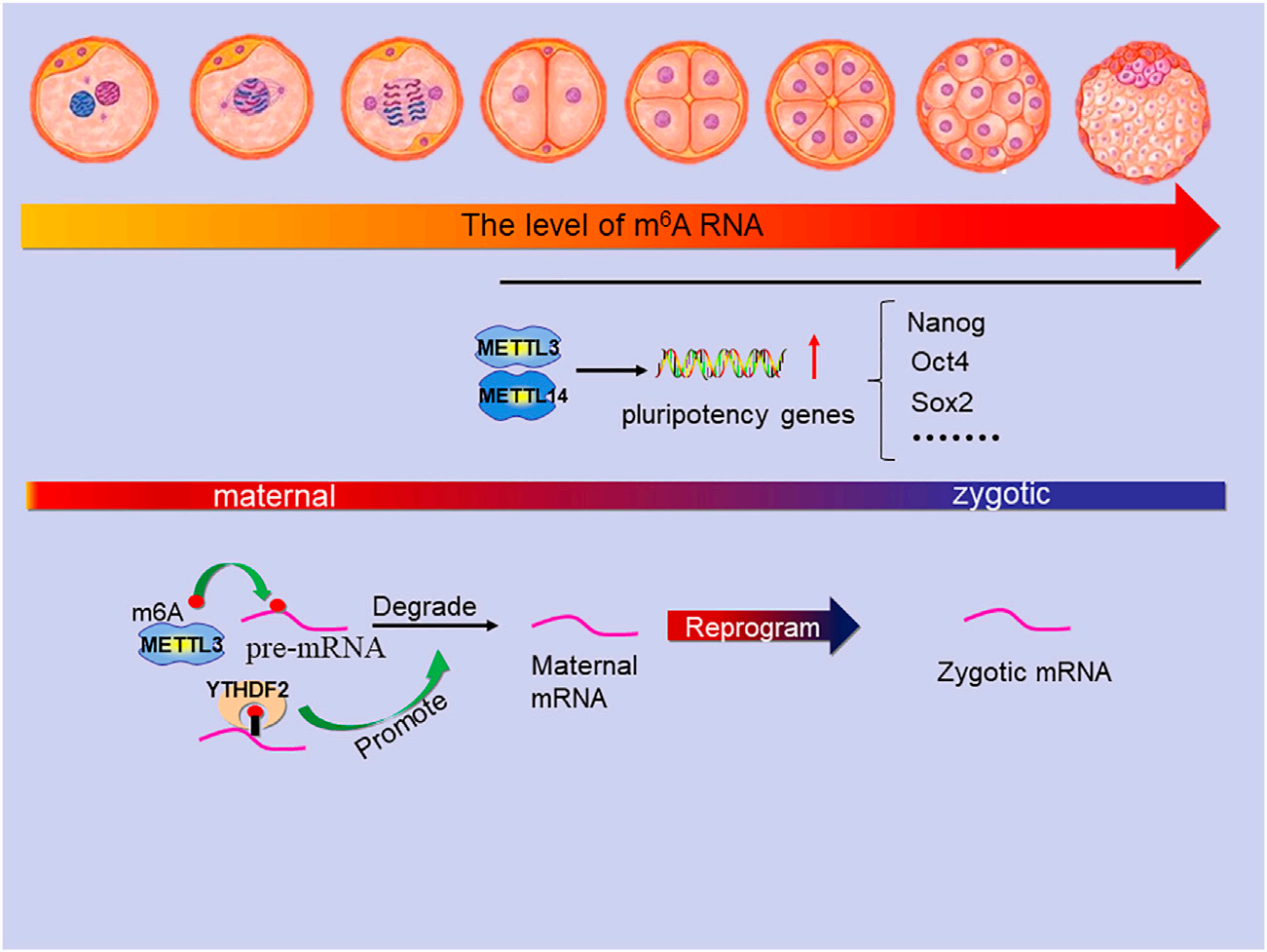
The main role of m^6^A in the stage of embryonic development. The level of m6A modification increased slowly in mouse 2-cell, 4-cell and 8-cell embryos but suddenly increased significantly in the transition period from morula to blastocyst which is due to the METTL3 and METTL14 active transcription of many pluripotency genes (includes Nanog, Oct4, Sox2 and so on). In the MZT, METTL3 mediates the m6A modification on pre-mRNA to degrade maternal mRNA and YTHDF2 can promote the process, which is reprogramming zygotic mRNA for zygotic development.

In mammals, fertilization of oocytes and sperm occurs in the ampulla of the fallopian tube. The fertilized egg enters the uterus along the fallopian tube and is implanted in the uterus (Figure 7). Usually, the main feature of the early stage embryo is the synchronous multiplication of the number of cells in the embryo, but after the 8-cell embryo stage, the embryonic cells gradually begin to divide asynchronously. This phenomenon is closely related to maternal-to-zygotic transition (MZT), MZT refers to a transition period of early embryonic development, in which embryonic development realizes the transformation from maternal factor clearance to ZGA (zygotic genome activation). In mice, after sperm and egg fuse at fertilization, MZT is initiated, while the transcription of embryonic genome is initiated in the late 1-cell stage (minor ZGA) and strongly activated in the 2-cell and 4-cell stages (major ZGA) (Schulz and Harrison, 2019); In humans (Asami et al., 2021), ZGA begins at the 1-cell stage, while in pigs (Oestrup et al., 2009), it mainly occurs at the 4-8 cell stage. After the embryo develops into a morula, the cells in the embryo gradually begin to differentiate into different types of cells. The inner non-polar cell subsets of morula will preferentially form inner cell mass (ICM), while the outer polar cells will develop into trophectoderm, participate in the initial contact and infiltration with the uterine wall, and finally form the placenta. The implanted embryo develops continuously to produce the fetus and its affiliated tissues. The embryonic cells differentiate into three germ layers: ectoderm, mesoderm and endoderm.

During the preimplantation period of mouse embryos, the m^6^A modification level showed dynamic changes, and m^6^A expression was higher in the blastocyst stage than in the 2-cell, 4-cell, and 8-cell stages (Faulds et al., 2018; Hao et al., 2019). Studies showed that the expression of METTL3 played an important role in this m^6^A modification (Kwon et al., 2019). In general, the level of m^6^A modification increased slowly in mouse 2-cell, 4-cell and 8-cell embryos; Compared with 2-cell embryos, blastocyst embryos showed a significant increase in m^6^A modification due to the active transcription of many genes. In pigs, it was found that m^6^A methylation continued from zygote stage to blastocyst stage, and the methylation level suddenly increased significantly in the transition period from morula to blastocyst. The *in vitro* application of cycloleucine (methylation inhibitor) effectively reduced the m^6^A modification level, significantly reduced the ratio of 4-cell embryos to blastocysts, and disturbed the normal embryonic cell differentiation (Yu et al., 2021). Interestingly, studies have shown that METTL3 mediated m^6^A methylation negatively regulates autophagy to support porcine blastocyst development (Cao et al., 2021). In this context, studies have also confirmed that gene knockout WTAP leads to mammalian embryonic development defects (Ping et al., 2014).

During the transformation from morula to blastocyst, the knockout of METTL3 reduced the m^6^A modification level, and the cells in the embryo remained in an undifferentiated state, resulting in embryonic lethality at the implantation stage (Geula et al., 2015; Cao et al., 2021). METTL14 is essential for postimplantation embryo development by promoting the conversion from naive to primed state of the epiblast. The deletion of METTL14 resulted in obvious embryonic growth retardation of embryo as early as embryonic day (E)6.5 and failure to differentiate, which led to the death of embryos in early pregnancy (Geula et al., 2015; Meng et al., 2019). Notably, Knockout of METTL3 and METTL14 reduced the expression levels of many stem cell pluripotency genes, such as Oct4, Sox2 and Nanog, in preimplantation mouse embryos (Wang et al., 2014).

GSK-3 (Glycogen synthase kinase-3) activity plays an important role in pluripotent stem cells. Intriguingly, GSK-3 deletion or inhibition by 2i medium containing inhibitors PD0325901 and CHIR99021 (Ying et al., 2008) can significantly increase the expression level of eraser FTO, significantly reduce the m^6^A modification level of mouse ES cells and improve the self-renewal of naive pluripotent stem cells (Faulds et al., 2018).

As a member of hnRNPs, hnRNP A2/B1 also plays an important regulatory role in m^6^A modification (Wu et al., 2018). As a mediator of m^6^A modification, hnRNP family members can open RNA structure and regulate RNA protein interaction (Liu et al., 2015). In addition, hnRNPu is closely related to the RNA-binding protein IGF2BP1 (IGF-II mRNA binding protein 1) in cell epigenetic modification. The RNA-binding protein IGF2BP1 stabilizes the RNA by associating with the CRD (Coding Region instability Determinant) and hnRNPu is essential to ensure stabilization of the mRNA via the CRD. IGF2BP1 also regulates the level of m^6^A modification during mouse embryonic development (Weidensdorfer et al., 2009). In addition, the study also confirmed that hnRNP A2/B1 also plays a crucial role in gene transcription and ES cells differentiation regulated by METTL3 dependent m^6^A modification (Kwon et al., 2019). Zc3h13 is a zinc finger protein that plays an important role in regulating nuclear RNA m^6^A methylation. Zc3h13 forms a complex with intranuclear factors WTAP, Virilizer and Hakai, which are located in the nucleus, and then plays a role in regulating the m^6^A methylation modification of nuclear RNA. Upon Zc3h13 knockdown, the majority of WTAP, Virilizer, and Hakai translocate to the cytoplasm. Concurrently, the nuclear components of methyltransferases METTL3 and METTL14 decrease and inhibit the formation of m^6^A. The knockout of Zc3h13 in mouse ES cells significantly reduced the overall m^6^A level on mRNA, impaired self-renewal and triggered mouse ES cell differentiation. Thus, Zc3h13 plays a key role in anchoring WTAP, Virilizer and Hakai in the nucleus to promote m^6^A methylation and regulate mouse ES cell self-renewal (Wen et al., 2018). In addition, endogenous retroviruses (ERVs) are a large number of heterogeneous integrated retroviral sequences. The methylation of ERV mRNA is catalyzed by the complex of methyltransferase like METTL3-METTL14 protein. m^6^A RNA methylation can limit ERVs and maintain the integrity of species cell genome (Chelmicki et al., 2021).

The reader YTHDC1 is located in the nucleus and plays an important role in mouse embryonic development (Kasowitz et al., 2018). Inactivated YTHDC1 can lead to embryo death. Previous studies have shown that although YTHDC2 knockout mice have developed into adulthood, both male and female adult mice are infertile (Hsu et al., 2017). During embryonic development, when the reader binds to m^6^A modified RNA, the m^6^A modified transcript target is activated, and the loss of YTHDC2 expression will lead to abnormal m^6^A modification in mice (Wojtas et al., 2017). Therefore, YTH domain proteins maintain mRNA stability and translation through m^6^A modification during embryonic development. As mentioned above, developing embryos are initially guided by maternal gene products. Then, during MZT, developmental control is handed over to ZGA. m^6^A modification can affect embryo reprogramming by participating in the transcriptional mechanism of maternal factor clearance, thus affecting the stability of m^6^A RNA and promoting the degradation of maternal mRNA. YTHDF2 plays an important role in this process by accelerating the degradation of mRNA. Loss of expression of YTHDF2 does not clear maternal mRNA, but can lead to abnormal embryonic development (Zhao and He, 2017). In addition, m^6^A modification can promote the translation of zygotic key transcriptional activators during MZT (Zhao et al., 2017). YTHDC protein plays an indispensable role in the self-renewal and multi-directional differentiation potential of pluripotent stem cells. In ES cells, YTHDC1 is necessary for rRNA synthesis and 2-cell transcriptional program inhibition, and regulates the scaffold function of LINE1 RNA. In fact, detailed analysis shows that YTHDC1 can recognize m^6^A on LINE1 RNA in the nucleus and regulate the formation of LINE1-nucleolin partnership and the recruitment of KAP1 chromatin. In YTHDC1 deficient ES cells and ICM cells, the establishment of H3K9me3 in 2C-related Long Terminal Repeat (LTR) retrotransposons was interrupted, thereby increasing transcriptional activity (Chen C. et al., 2021). In fact, other members of YTHDF protein have different effects on the differentiation of ES cells. For example, in ES cells, the deletion of YTHDF3 leads to the loss of cell pluripotency and accelerates the expression of marker genes related to the formation of three germ layers. Phenotypic and transcriptomic analysis showed that the deletion of YTHDF1 seriously blocked cardiomyocyte differentiation, accompanied by the down-regulation of specific genes. On the contrary, YTHDF3 knockout accelerates differentiation by promoting the expression of cardiomyocyte specific genes. It is worth noting that YTHDF3 seems to regulate cell differentiation by inhibiting YTHDF1, supporting the opposite role of YTHDF1 and YTHDF3 in cell fate determination (Wang et al., 2020).

Finally, in the process of trophoblast invasion of the uterus tissues, Li et al. (2019) reported that in the experiment of villus explant culture, ALKBH5 knockout promoted the invasion of trophoblast cells, and the over-expression of ALKBH5 inhibited the invasion of trophoblast cells. In addition, ALKBH5 inhibits the invasion of trophoblast cells by regulating the stability of Cyr61 mRNA (Li et al., 2019).

## Conclusion

N- methyladenosine (mA) is a large number of modifications to mRNA and DNA. It was first discovered and characterized in the 1970s. Among them, m^6^A is a very common RNA modification in mRNA and non-coding RNA, which affects RNA splicing, translation and stability, as well as the epigenetic effects of some non-coding RNAs. The progress of the latest sequencing technology maps m^6^A to the transcriptome of cells in various model systems. At the same time, we are gradually mastering the function of m^6^A modification and the key regulators of methylation and demethylation, such as the readers (m^6^A binding proteins), writers (methyltransferases) and erasers (demethylases). It is now clear that expression and activities of these proteins are essential for the correct regulation of most if not all reproductive processes. Strong evidence has emerged on the various functions of these proteins and the corresponding functions of targeted RNA in oocyte, sperm and embryonic development. The mTORC1 signaling pathway is activated in granulosa cells and oocytes during the process of follicular activation and m6A RNA modification is closely related to the mTORC1 signaling pathway of cancer cells (Chen H. et al., 2021). These are just two areas amongst many others that are worthy of in-depth investigation and discussion in the future. In addition, no information is available about m^6^A functions in the early stages of gametogenesis including the formation of the primordial germ cells (the precursors of gametes) and sex differentiation of the germ cells and somatic cells of the gonads. Interestingly, in this regard, in *Drosophila*, IME4 (a homologous of METTL3)-null mutants show a sex bias towards males.

Of particular interest is the role of m^6^A modification in development and reprogramming during MZT and ZGA. In addition, the wrong m^6^A RNA modification during embryonic development impacts the differentiation process, which is worthy of further study. With the advancement of technology, such as gene-editing technology and the continuous updating of various detection technologies, it is likely that the understanding of the regulation of m^6^A in reproductive and developmental processes will continue to improve in the years to come.
